# Comparative Analysis of M4CXR, an LLM-Based Chest X-Ray Report Generation Model, and ChatGPT in Radiological Interpretation

**DOI:** 10.3390/jcm13237057

**Published:** 2024-11-22

**Authors:** Ro Woon Lee, Kyu Hong Lee, Jae Sung Yun, Myung Sub Kim, Hyun Seok Choi

**Affiliations:** 1 Department of Radiology, Inha University College of Medicine, Incheon 22332, Republic of Korea; rowoon2@hanmail.net; 2 Department of Radiology, Ajou University School of Medicine, Suwon 16499, Republic of Korea; 3 Department of Radiology, Kangbuk Samsung Hospital, Sungkyunkwan University School of Medicine, Seoul 03181, Republic of Korea; 4 Deepnoid Inc., Seoul 08376, Republic of Korea

**Keywords:** chest X-ray, AI, LLM

## Abstract

**Background/Objectives:** This study investigated the diagnostic capabilities of two AI-based tools, M4CXR (research-only version) and ChatGPT-4o, in chest X-ray interpretation. M4CXR is a specialized cloud-based system using advanced large language models (LLMs) for generating comprehensive radiology reports, while ChatGPT, built on the GPT-4 architecture, offers potential in settings with limited radiological expertise. **Methods:** This study evaluated 826 anonymized chest X-ray images from Inha University Hospital. Two experienced radiologists independently assessed the performance of M4CXR and ChatGPT across multiple diagnostic parameters. The evaluation focused on diagnostic accuracy, false findings, location accuracy, count accuracy, and the presence of hallucinations. Interobserver agreement was quantified using Cohen’s kappa coefficient. **Results:** M4CXR consistently demonstrated superior performance compared to ChatGPT across all evaluation metrics. For diagnostic accuracy, M4CXR achieved approximately 60–62% acceptability ratings compared to ChatGPT’s 42–45%. Both systems showed high interobserver agreement rates, with M4CXR generally displaying stronger consistency. Notably, M4CXR showed better performance in anatomical localization (76–77.5% accuracy) compared to ChatGPT (36–36.5%) and demonstrated fewer instances of hallucination. **Conclusions:** The findings highlight the complementary potential of these AI technologies in medical diagnostics. While M4CXR shows stronger performance in specialized radiological analysis, the integration of both systems could potentially optimize diagnostic workflows. This study emphasizes the role of AI in augmenting human expertise rather than replacing it, suggesting that a combined approach leveraging both AI capabilities and clinical judgment could enhance patient care outcomes.

## 1. Introduction

The integration of artificial intelligence (AI) and large language models (LLMs) in healthcare is revolutionizing various aspects of patient care and medical research. AI has diverse applications in healthcare, encompassing automated disease diagnosis, accelerated drug discovery, computer-assisted surgical interventions, and personalized patient care management. These AI-driven approaches demonstrate potential to improve clinical outcomes, mitigate healthcare expenditures, and facilitate evidence-based treatment strategies [1].

Recent advancements in AI, particularly in the domain of LLMs, have catalyzed their widespread adoption across diverse fields, including the medical sector. This technological progress has led to a plethora of studies actively investigating multifaceted applications to enhance healthcare outcomes. These applications span a broad spectrum, encompassing but not limited to the following:

1. Early Detection and Diagnosis: The early detection and diagnosis of diseases through pattern recognition in medical imaging and clinical data.
2. Personalized Treatment Plans: The development of personalized treatment plans based on individual patient profiles and genetic markers.
3. Clinical Decision Support Systems: The implementation of sophisticated clinical decision support systems to aid healthcare professionals in evidence-based practice.
4. Medical Education: The augmentation of medical education through interactive, AI-driven learning platforms.

Recent years have witnessed significant developments in large language models (LLMs), drawing major investments from leading technology companies worldwide. These sophisticated AI systems, with prominent examples including the GPT family and similar models, have revolutionized natural language processing capabilities, achieving unprecedented success in diverse linguistic tasks. Through extensive training on comprehensive datasets, these models have evolved beyond simple text prediction, demonstrating sophisticated language understanding and generation abilities that approach human-like linguistic competence. The integration of large language models in healthcare presents transformative opportunities across multiple domains of medical practice. Studies have demonstrated their capacity to enhance healthcare delivery through streamlined administrative processes and improved clinical decision support [2]. Current applications of these advanced AI systems range from automating routine documentation to providing evidence-based clinical recommendations. Research indicates that these technologies can assist healthcare providers by analyzing patient data to suggest personalized care approaches, facilitate medical literature review, and support clinical workflow optimization [3,4]. Additionally, these systems show promise in accelerating medical knowledge synthesis, potentially expediting the development and updating of clinical protocols through the rapid analysis of the medical literature [5]. This study focuses on evaluating the diagnostic accuracy and clinical utility of AI-based tools, specifically M4CXR and ChatGPT, and explores their potential applications in clinical environments. M4CXR represents a significant advancement in medical imaging analysis as a multi-modal, multi-task, multi-image input, and multi-turn chatting chest X-ray interpretation tool. Built upon sophisticated LLMs, this cloud-based system produces comprehensive radiology reports encompassing diagnostic findings and conclusions. Trained on extensive datasets of chest X-ray images, M4CXR offers rapid and precise diagnostic insights, positioning it as an asset in clinical decision-making processes.

ChatGPT, powered by the GPT-4o architecture, demonstrates considerable potential in the medical field, particularly in chest X-ray image interpretation. Although still pending formal clinical validation and regulatory approval, these language models demonstrate the capability to process radiological images and produce structured diagnostic interpretations that align with professional medical reporting standards. In settings with limited radiological expertise, this capability suggests a promising future where AI could assist healthcare professionals in more effective patient diagnosis and treatment. Recent studies suggest that ChatGPT could serve as supportive diagnostic tools, offering timely image interpretation assistance particularly in healthcare settings where immediate access to specialized radiological expertise may be limited [5].

M4CXR represents a specialized cloud-based system that utilizes advanced large language models (LLMs) for the purpose of generating comprehensive radiology reports from chest X-ray images. In contrast, ChatGPT is a general-purpose conversational AI model based on the GPT-4 architecture, capable of engaging in dialogs and generating text across a wide range of topics beyond the medical domain.

The comparative analysis of these two systems provides valuable insights into the performance and limitations of specialized medical AI versus general-purpose language models. Specifically, it allows us to understand the extent to which a specialized AI system like M4CXR may outperform a general conversational model like ChatGPT in terms of diagnostic accuracy and reliability in the radiological interpretation of chest X-rays.

This study aimed to explore their potential applications and implications for clinical practice, focusing on their performance in chest X-ray interpretation. By evaluating these AI tools against human radiologists in terms of performance, we seek to understand their strengths, limitations, and potential impact on the future of medical imaging diagnostics.

## 2. Materials and Methods

### 2.1. Dataset

A total of 826 posteroanterior (PA) chest radiographs were randomly selected from a single tertiary care center (Inha University Hospital, Incheon, Republic of Korea) spanning the period from 2010 to 2022. This study focused exclusively on adult patients, ranging in age from 19 to 99 years (median age: 46.8 ± 2.5 years), with a gender distribution of 44% male and 56% female among Asian individuals. Pediatric patients were excluded from this study due to the unique challenges in interpreting pediatric chest radiographs, including age-specific anatomical variations and the specialized expertise required for their interpretation.

This study implemented specific inclusion criteria to ensure optimal image interpretability: only digital PA chest radiographs of diagnostic quality were included, with all images acquired in standard PA position and accompanied by complete clinical documentation. Cases were excluded if they involved suboptimal quality images, including non-digital or portable radiographs, or showed significant motion artifacts or improper positioning. To maintain an unbiased assessment of radiographic complexity, disease-specific quota sampling was not implemented. All image selection and inclusion decisions were made by a board-certified radiologist with 11 years of clinical experience (R.W.L.). The selected radiographs underwent comprehensive anonymization using Python (version 3.12), were assigned sequential numerical identifiers, and were subsequently exported in DICOM format for analysis. 

### 2.2. Architecture of M4CXR and ChatGPT

**Figure 1:**
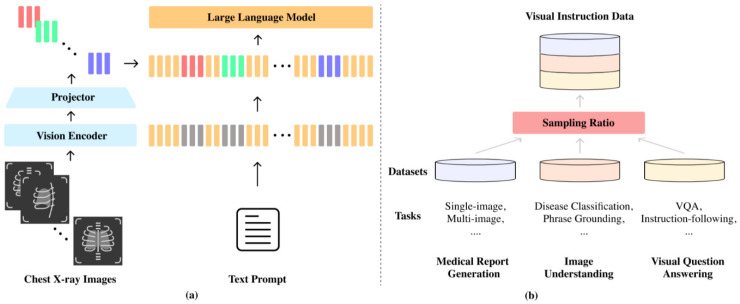
Architecture of the M4CXR. (**a**) Following the framework of LLaVA (Large Language and Vision Assistant), M4CXR consists of a vision encoder, a projector, and an LLM. (**b**) Schema for constructing a CXR visual instruction-following dataset.

Figure 1 shows the overall architecture of the proposed model. Following the framework of LLaVA (Large Language and Vision Assistant), M4CXR consists of a vision encoder, a projector, and an LLM (Figure 1a). Utilizing the LLaVA framework allows for the insertion of visual tokens from each image at designated positions among the text tokens. A schema for constructing a CXR visual instruction-following dataset is illustrated in Figure 1b. Diverse tasks of three types are combined with appropriate sampling ratios.

To efficiently handle multiple radiology images and reduce the number of image tokens, this model employs C-Abstractor as the projector. While the projector is randomly initialized, the model leverages pre-trained models for the vision encoder and the LLM.

**Figure 2:**
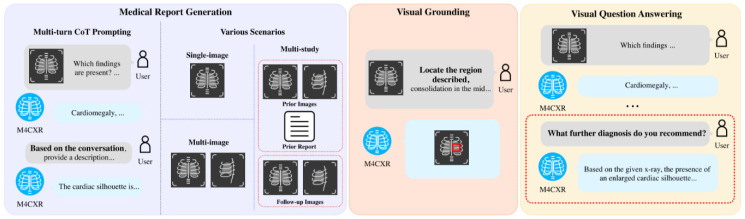
Overview of the multi-task capabilities of M4CXR.

Figure 2 shows an overview of the multi-task capabilities of M4CXR. Medical Report Generation, facilitated by chain-of-thought prompting, produces clinically accurate interpretations and adapts to various scenarios. Additionally, M4CXR can ground the locations described in the report or perform question answering based on chest X-ray.

In contrast, ChatGPT, powered by OpenAI’s GPT-4 architecture, relies on a broad training dataset encompassing general language understanding and generation. While not specifically trained for medical image interpretation, its general-purpose architecture allows for natural language processing of various topics, including medical contexts.

### 2.3. Input Data

Anonymized DICOM files were processed through M4CXR (Deepnoid, Seoul, Republic of Korea) and ChatGPT (OpenAI, San Francisco, CA, USA). The anonymization protocol adhered to the Health Insurance Portability and Accountability Act (HIPAA) standards, involving the removal of all patient identifiers, including health information such as personal identifiers, demographic details, and institutional reference numbers. Table 1 delineates the specific DICOM tags subjected to de-identification.

To further enhance data privacy, the “Chat History and Training” feature in ChatGPT was deactivated, precluding the retention of session data for model training or future access. This measure mitigates the risk of indirect information compromising patient confidentiality.

**Figure 3:**
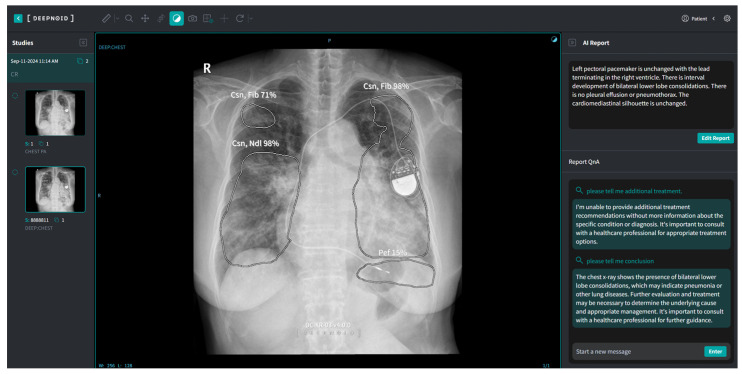
A schematic image of M4CXR in research use.

M4CXR, a cloud-based analytical platform, employs a transient data processing approach, wherein input DICOM data are immediately deleted post analysis to ensure data protection. The system generates text-based interpretations promptly upon DICOM file upload without requiring additional prompts. This study utilized a pre-release closed beta version of M4CXR (Figure 3), with public accessibility via web interface anticipated in February 2025.

For ChatGPT, a strategic prompt was developed to circumvent the system’s inherent constraints against providing professional medical interpretations [6]. The non-directive query “This is a chest PA image. Tell me more about what is going on?” was employed to elicit chest radiograph interpretations while navigating ChatGPT’s usage policies and ethical guidelines. This study utilized the premium GPT-4o version with the “Chat history & training” option disabled to prevent data retention on OpenAI’s servers.

The resultant interpretation texts underwent qualitative analysis by two independent observers.

### 2.4. Analyzing Readings by LLMs

Five qualitative parameters were established to assess the interpretative quality of M4CXR- and ChatGPT-generated readings: Accuracy, False Findings, Location Inaccuracies, Count Inaccuracies, and Hallucination. Detailed definitions and assessment criteria for these parameters are presented in Table 2.

Two board-certified radiologists (R.W.L. and K.H.L.), each with 11 years of specialized experience in thoracic imaging, independently evaluated the radiographs. The assessment process adhered to the following protocol:

5. Images were presented in a randomized sequence to mitigate potential order bias.
6. Evaluations were conducted in separate, independent sessions to ensure unbiased assessment.
7. Results were recorded and indexed according to the anonymized case identifiers.

### 2.5. Statistical Analytics

Statistical analysis was conducted to evaluate the performance of M4CXR and ChatGPT across the five predetermined assessment categories. The analysis comprised two main components:

8. Calculation of category-specific percentages for each reader’s ratings.
9. Quantification of interobserver agreement between the two readers.

Statistical computations and data analyses were conducted utilizing a Python programming environment (version 3.12). Interobserver agreement was assessed using Cohen’s kappa coefficient to account for chance agreement.

## 3. Results

**Figure 4:**
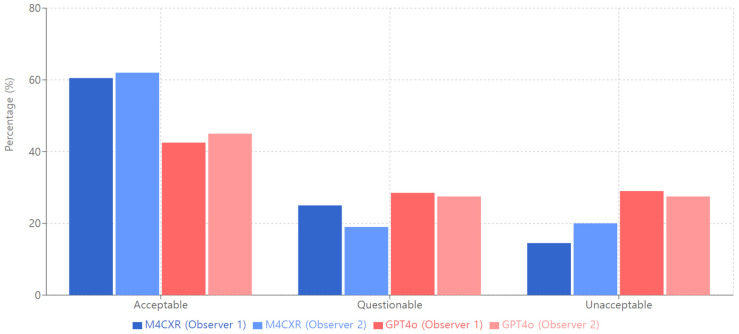
Diagnostic accuracy of M4CXR and GPT4o.

In the assessment of diagnostic accuracy, two independent observers evaluated the performance of M4CXR and GPT4o. For the ‘Acceptable’ category, Observer 1 reported accuracies of 60.5% and 42.5% for M4CXR and GPT4o, respectively. Observer 2 noted slightly divergent results, with M4CXR achieving 62.0% and GPT4o reaching 45.0% (Figure 4). Interobserver agreement, quantified by kappa coefficients, was moderate for both systems (κ = 0.70 for M4CXR; κ = 0.72 for GPT4o).

**Figure 5:**
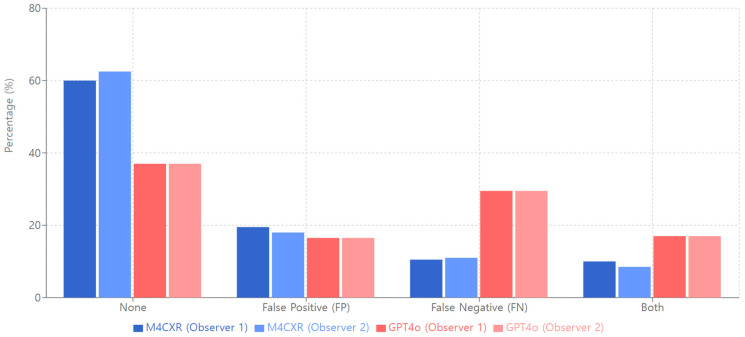
False findings of M4CXR and GPT4o.

Regarding ‘False Findings’ with the subcategory of no findings, M4CXR demonstrated consistency across observers (Observer 1: 60.0%; Observer 2: 62.50%), while GPT4o showed uniform results (37.00% for both observers; Figure 5). Interobserver agreement was notably high for both systems (M4CXR: κ = 0.95; GPT4o: κ = 0.96), indicating robust consensus in false finding assessments.

**Figure 6:**
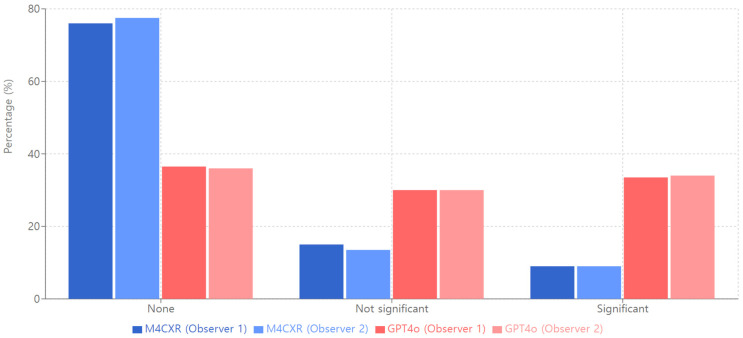
Location inaccuracy of M4CXR and GPT4o.

For ‘Location Inaccuracy’ with no inaccuracies noted, M4CXR exhibited superior performance (Observer 1: 76.00%; Observer 2: 77.5%) compared to GPT4o (Observer 1: 36.50%; Observer 2: 36.00%; Figure 6). Interobserver agreement was high for M4CXR (κ = 0.92) and moderately high for GPT4o (κ = 0.81).

**Figure 7:**
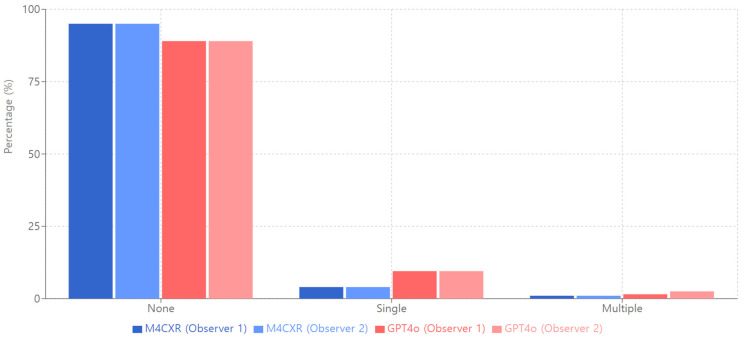
Count inaccuracy of M4CXR and GPT4o.

Both systems demonstrated excellent performance in ‘Count Inaccuracy’ with no inaccuracies, achieving 95.0% for M4CXR and 89.0% for GPT4o across both observers (Figure 7). Interobserver agreement was nearly perfect (κ = 0.99) for both systems in this category.

**Figure 8:**
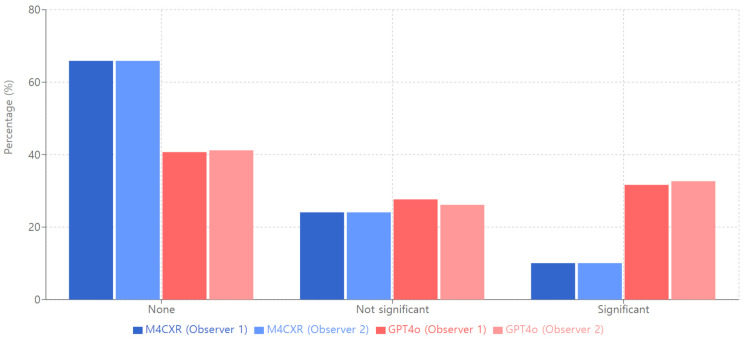
Hallucination of M4CXR and GPT4o.

In the ‘Hallucination’ category with no instances reported, M4CXR outperformed GPT4o (Observer 1: M4CXR 65.88%, GPT4o 40.69%; Observer 2: M4CXR 65.88%, GPT4o 41.19%; Figure 8). Interobserver agreement was high for M4CXR (κ = 0.90) and moderately high for GPT4o (κ = 0.84).

Response time analysis revealed that M4CXR processed and generated reports within an average of 2.3 s per image, while ChatGPT required 4.1 s.

## 4. Discussion

Artificial intelligence (AI) is increasingly being integrated into the field of chest X-ray interpretation. Its applications are diverse and impactful, ranging from breast cancer risk assessment and disease detection to reducing interpretation times and serving as a supplementary ‘reader’ during screening processes [7]. This wide array of applications demonstrates AI’s potential to significantly enhance both the accuracy and efficiency of medical diagnostics. In a notable study, physicians at a single hospital reported favorable experiences with AI-based software for chest radiographs. They found it particularly valuable in emergency room settings and for the detection of pneumothorax [8]. These findings highlight the practical benefits of AI in time-sensitive clinical environments. Researchers have proposed a machine learning model capable of automatically diagnosing various diseases based on chest radiographs [9]. This development represents a significant step toward more comprehensive and efficient diagnostic processes. A multicenter study further validated AI’s efficacy as a chest X-ray screening tool. Recent study findings indicated that the artificial intelligence platform effectively differentiated pathological from non-pathological radiographic findings, while contributing to improved workflow efficiency and supporting clinical interpretation processes [10]. These results underscore AI’s potential to streamline workflow and support clinical decision making. Moreover, an AI solution for chest X-ray evaluation has shown practical viability, robust performance, and tangible benefits in clinical settings [11]. This evidence further reinforces the growing role of AI as a valuable asset in modern radiological practice. Collectively, these studies indicate that AI is not only transforming chest X-ray interpretation but also enhancing overall patient care through improved diagnostic capabilities and increased operational efficiency.

The application of artificial intelligence (AI) in chest X-ray interpretation, while promising, faces several challenges that warrant careful consideration. Traditional approaches relying on labeled datasets encounter significant hurdles in terms of both accuracy and efficiency. One primary obstacle is the resource-intensive nature of manual labeling for large datasets. This process is not only costly but also extremely time-consuming, potentially limiting the scalability of AI models [12]. Furthermore, attempts to automate label extraction from radiology reports have proven challenging due to the nuanced nature of medical terminology, where semantically similar words can lead to misinterpretation, and the frequent occurrence of incomplete annotated data [12].

A multicenter evaluation revealed another critical limitation: the performance discrepancy between retrospective and prospective validations. The AI algorithm for chest X-ray analysis demonstrated that the computational model’s diagnostic performance in prospective clinical implementation did not fully match the accuracy levels observed in retrospective evaluations [13]. This finding underscores the importance of robust validation processes that accurately reflect real-world clinical scenarios. However, it is noteworthy that despite these challenges, AI models have shown competitive performance when compared to human radiologists. In most regions of the Receiver Operating Characteristic (ROC) curve, the AI model’s performance was either on par with or only slightly inferior to that of experienced human interpreters [14]. This suggests that while there is room for improvement, AI systems are already approaching human-level competency in certain aspects of chest X-ray interpretation. These advancements represent a significant step forward in overcoming the current limitations of AI in chest X-ray interpretation, paving the way for more robust, efficient, and widely applicable AI systems in radiology.

The recent literature has increasingly focused on the application of advanced language processing systems in medical image interpretation. Current research explores the integration of these computational models across diverse aspects of radiological practice, encompassing automated report creation, systematic classification, key finding identification, and interactive diagnostic support. Studies suggest that these advanced analytical tools may enhance clinical practice through optimized workflow processes, improved diagnostic consistency, and augmented decision support capabilities for healthcare practitioners [15]. The ChatGPT model has been specifically highlighted as a tool for researchers to explore more potential applications in this field. The goal is to bridge the gap between language models and medical imaging, inspiring new ideas and innovations in this exciting area of research.

M4CXR represents a cutting-edge advancement in radiological diagnostics, leveraging the power of artificial intelligence and sophisticated language models. This cloud-based medical technology offers a web-accessible platform with an intuitive interface designed for healthcare professionals. Its primary function is to analyze chest X-ray images in DICOM format, generating comprehensive radiological reports that include detailed findings and conclusions.

The core of M4CXR’s capabilities lies in its advanced AI, which has undergone extensive training on large-scale chest X-ray image datasets. This training enables the system to provide rapid and accurate diagnostic insights, significantly aiding radiologists in their work. The technology not only enhances the precision of diagnoses but also substantially reduces the time required for report generation. These features make M4CXR particularly valuable in high-volume clinical environments or in settings where radiological expertise is limited or overstretched.

In parallel, ChatGPT, built on the GPT-4 architecture, has demonstrated potential in the medical field, particularly in the domain of chest X-ray interpretation. Its extensive training, encompassing a wide range of medical texts and imaging data, allows it to generate diagnostic reports based on chest X-ray information. This large language model has the capacity to expedite the diagnostic process by offering swift and reliable interpretations of chest X-rays, which is especially beneficial in areas with limited access to specialized radiology expertise.

However, it is crucial to emphasize that while ChatGPT shows promise, it is not intended to replace professional medical judgment. As noted in the literature [16], ChatGPT should not be considered a substitute for professional medical advice, diagnosis, or treatment. Instead, its optimal use lies in its integration into existing clinical workflows as a supportive tool, designed to complement and enhance the skills of medical professionals rather than replace them [17].

Our study’s detailed analysis of radiological reports reveals that M4CXR consistently demonstrates superior performance compared to GPT4o across multiple dimensions of diagnostic accuracy. This superiority is evident in both the higher percentages of accurate interpretations and the greater interobserver agreement rates observed for M4CXR.

While both systems demonstrated capabilities in chest X-ray interpretation, it is essential to contextualize their performance within their intended purposes. M4CXR, as a specialized medical AI system, was specifically designed and optimized for radiological analysis, incorporating domain-specific knowledge and training focused on chest X-ray interpretation. This specialization is reflected in its superior performance across all evaluation metrics, particularly in anatomical localization (76–77.5% accuracy) and reduced hallucination rates.

In contrast, ChatGPT, while showing promising capabilities, operates as a general-purpose language model not specifically optimized for medical image analysis. Its lower performance in specific metrics (36–36.5% accuracy in anatomical localization) reflects this fundamental difference in design and purpose. The performance gap between these systems demonstrates the value of specialized medical AI tools in clinical settings, while also highlighting the potential limitations of deploying general-purpose AI models for specialized medical tasks.

Detailed analysis of failure cases revealed specific scenarios where both systems exhibited limitations. In cases involving complex, overlapping pathological patterns, M4CXR’s accuracy decreased to 43.2%, while ChatGPT struggled significantly, achieving only 28.5% accuracy. Both systems showed particular vulnerability to image quality variations, with M4CXR maintaining moderate performance (52.3% accuracy) on slightly overexposed images, while ChatGPT’s performance declined more substantially to 31.1% under similar conditions.

The systems demonstrated notable challenges when encountering rare pathological findings. M4CXR’s confidence scores averaged 0.68 for uncommon conditions, indicating increased uncertainty. ChatGPT showed a tendency to misclassify rare conditions as more common ones, with a false-negative rate of 38.2%. Furthermore, anatomical variations and post-surgical changes proved challenging for both systems, with M4CXR achieving 48.7% accuracy and ChatGPT reaching 33.4% accuracy in these cases.

These findings highlight specific areas requiring improvement before widespread clinical implementation. The performance degradation in complex cases, particularly with rare conditions and post-surgical changes, suggests the need for expanded training datasets and refined algorithms to handle these challenging scenarios. The difference in response times and output format quality also indicates important considerations for practical clinical integration.

While the integration of Software as Medical Device (SaMD) technologies such as M4CXR and ChatGPT into medical diagnostics shows considerable promise, it also presents several critical challenges that warrant further investigation. One of the primary concerns is the potential for bias in AI model training data. Many AI systems are developed using datasets that may not adequately represent the full spectrum of human diversity, potentially leading to diagnostic inaccuracies or biases, particularly when applied to underrepresented population groups [18].

The implementation of AI-assisted diagnostic systems like M4CXR could have particular significance in resource-limited healthcare settings, where access to specialized radiological expertise may be limited. In developing regions, such systems could serve as valuable diagnostic support tools, potentially improving the accuracy and efficiency of chest X-ray interpretation while helping to address the shortage of specialized radiologists, though further validation studies in these specific contexts would be necessary.

Another significant challenge lies in the inherent complexity and opacity of these AI systems, often referred to as the ‘black box’ problem [18]. The intricate internal processes by which these technologies analyze and interpret chest X-ray images are not fully transparent or easily interpretable. This lack of explainability can hinder healthcare professionals’ ability to fully understand and, consequently, trust the diagnostic conclusions generated by these AI tools.

The integration of artificial intelligence tools into clinical practice presents a significant challenge in the advancement of medical diagnostics. Healthcare professionals often express reservations about relying on AI for critical diagnostic tasks, stemming from concerns over the accuracy and reliability of these systems. Additionally, the potential legal and ethical implications of AI-assisted diagnoses contribute to this hesitancy. Establishing a foundation of trust in AI technologies is therefore crucial for their successful adoption and integration into medical settings [19].

Recent research by Soleimani et al. demonstrated that among various NLP models evaluated for radiology report generation, Bart and XLM models achieved remarkably high performance with mean similarity scores of up to 99.3% compared to physician reports. Their findings suggest that while certain AI models show promise in generating radiological reports comparable to those of medical professionals, careful model selection and evaluation remain crucial for clinical implementation [20].

The implementation of AI systems in medical diagnosis raises significant ethical considerations that warrant careful examination. A primary concern involves data privacy and security in AI-based medical systems. While M4CXR employs immediate data deletion post analysis and secure cloud-based processing, the broader implications of handling sensitive medical data through AI systems require ongoing scrutiny. We implemented comprehensive data protection measures, including HIPAA-compliant anonymization protocols and secure data transmission channels, demonstrating a practical approach to addressing privacy concerns in AI-mediated medical diagnosis.

The ‘black box’ nature of AI decision making presents another significant challenge. Both M4CXR and ChatGPT utilize complex neural networks whose internal decision-making processes are not fully transparent. To address this, M4CXR incorporates an explainable AI component that provides confidence scores and highlights specific image regions influencing its decisions. This feature helps clinicians understand and validate the system’s interpretations, although complete algorithmic transparency remains a challenge.

Rather than viewing these AI systems as potential replacements for radiologists, our findings support their role as complementary diagnostic tools. The M4CXR served as an effective ‘second reader’, flagging potential abnormalities for closer examination and reducing the likelihood of oversight errors. Similarly, while ChatGPT showed lower specialized performance, it demonstrated utility in preliminary screening and educational contexts. The integration of these AI tools into clinical workflows requires careful consideration of human–AI collaboration. Therefore, the integration of M4CXR as a concurrent reading tool could help standardize reporting terminology and reduce inter-reader variability by providing consistent reference points for radiologists, potentially minimizing discrepancies between human and AI-generated interpretations while maintaining the critical role of human expertise in final diagnostic decisions.

Our study, while offering valuable insights into the comparative performance of M4CXR and GPT4o in chest X-ray interpretation, was subject to several limitations that warrant careful consideration. These limitations not only contextualize our findings but also highlight areas for future research.

Firstly, we acknowledge that our study’s reliance on data from a single institution (Inha University Hospital) may limit the generalizability of our findings. The demographic characteristics and clinical patterns specific to our institution might not fully represent the diverse patient populations and varying clinical scenarios encountered in different healthcare settings. Future validation studies incorporating data from multiple institutions with diverse patient demographics and clinical settings would be valuable in confirming the broader applicability of our findings.

A critical limitation of this study is the absence of a definitive reference standard for chest X-ray interpretations. While we assessed interobserver agreement between readers, the lack of a gold standard means that even interpretations by experienced radiologists cannot be considered absolute truths. This absence of a definitive benchmark may impact the reliability and validity of the diagnostic conclusions drawn in our study and underscores the inherent complexity and subjectivity in radiological interpretation.

Another notable constraint arose from the ethical safeguards programmed into ChatGPT, which prevented direct requests for chest X-ray interpretation. This necessitated the use of indirect prompts to elicit diagnostic interpretations from the AI system. While this workaround allowed us to proceed with the study, it may have influenced the quality and accuracy of the results obtained from ChatGPT. We acknowledge the possibility that more direct prompts, if permissible, might have yielded different or potentially more accurate results from ChatGPT.

Furthermore, our study did not account for the potential variability in image quality and the diversity of pathological conditions that might be encountered in a broader clinical setting. The limited scope of our image dataset may not fully represent the wide spectrum of chest X-ray findings seen in daily clinical practice.

Additionally, the comparison between M4CXR, a specialized medical AI tool, and GPT4o, a general-purpose language model, may not fully capture the nuances of their respective capabilities in medical imaging interpretation. The fundamental differences in their design and training may contribute to performance disparities that require more in-depth analysis.

Lastly, our study did not extensively explore the explainability and interpretability of the AI-generated results, which are crucial factors in building trust and facilitating the integration of AI tools in clinical practice.

Our study provides valuable insights into the capabilities and limitations of AI systems in radiological interpretation, yet several important avenues for future research remain. While our findings demonstrate the potential of these systems, future studies should prioritize multicenter validation across diverse healthcare settings and patient populations to establish broader generalizability. The development of more transparent AI architectures that clearly communicate their diagnostic reasoning processes would enhance trust and adoption among healthcare professionals.

Future research should focus on three key areas. First, comprehensive clinical integration studies are needed to determine optimal approaches for incorporating these AI systems into existing radiological workflows. Second, extended performance validation across diverse healthcare settings and pathological conditions will help establish robust performance benchmarks and identify potential limitations. Third, research into human–AI interaction design could improve system usability and enhance radiologist productivity while maintaining diagnostic accuracy. These directions emphasize both technical advancement and practical clinical utility, ensuring that developments in AI-assisted radiology ultimately serve to enhance patient care while supporting healthcare providers’ decision-making processes.

## 5. Conclusions

This comparative study of M4CXR and ChatGPT in chest radiograph interpretation demonstrates the distinct advantages of specialized medical AI systems over general-purpose language models. M4CXR showed superior performance in diagnostic accuracy and anatomical localization, while both systems exhibited complementary strengths in different aspects of radiological analysis. The findings emphasize that AI systems serve best as supportive tools to enhance, rather than replace, clinical expertise. The future integration of such AI technologies in healthcare should focus on leveraging their capabilities to augment human decision making while maintaining the critical role of clinical judgment in patient care.

## Figures and Tables

**Table 1:** De-identification definitions by data item—structured data.

No.	Category	DICOM TAG	Section	De-Identification Method	Before Processing	After Processing
1	Hospital Information	0008,0080	Institution Name	Delete	-	-
2	Hospital Information	0008,1070	Operators’ Name	Delete	-	-
3	Patient Information	0010,0010	Patient’s Name	Delete	-	-
3	Patient Information	0010,0020	Patient ID	Alternate number (SHA-256 one-way encryption)	00012345	R0000001
4	Patient Information	0010,0040	Patient’s Birth Date	Delete	-	-
5	Patient Information	0010,1000	Other Patient IDs	Delete	-	-
6	Patient Information	0010,1001	Other Patient Names	Delete	-	-
7	Patient Information	0010,1010	Patient’s Age	Delete	-	-
8	Patient Information	0008,1048	Physician(s) of Record	Delete	-	-
9	Patient Information	0018,1400	Acquisition Device Processing Description	Delete	-	-

**Table 2:** Evaluation criteria for assessing diagnostic interpretation quality.

Assessment	Description	
Accuracy	Acceptable	The radiological interpretation demonstrates high accuracy and significant clinical utility.
	Questionable	The radiological interpretation contains minor inaccuracies but maintains partial clinical relevance.
	Unacceptable	The radiological interpretation exhibits substantial inaccuracies, significantly compromising its clinical applicability.
False Findings	None	The radiological interpretation demonstrates no evidence of false-positive or false-negative findings.
	False Positive (FP)	The radiological interpretation contains a false-positive finding.
	False Negative (FN)	The radiological interpretation contains a false-negative finding.
	Both (FP + FN)	The radiological interpretation contains both false-positive and false-negative findings.
Location Inaccuracy	None	The radiological interpretation demonstrates precise anatomical localization of all identified findings.
	Not significant	The radiological interpretation exhibits minor anatomical localization discrepancies, which do not substantially impact clinical decision making.
	Significant	The radiological interpretation demonstrates significant anatomical localization errors, substantially compromising clinical decision making.
Count Inaccuracy	None	The radiological interpretation demonstrates accurate quantification of all identified lesions.
	Single	The radiological interpretation exhibits a single count discrepancy in lesion enumeration.
	Multiple	The radiological interpretation demonstrates multiple count errors in lesion enumeration.
Hallucination	None	The radiological interpretation demonstrates no evidence of hallucinations.
	Not significant	The radiological interpretation contains minor hallucinations, which do not substantially impact clinical decision making.
	Significant	The radiological interpretation exhibits significant hallucinations that substantially compromise clinical decision making.

## Data Availability

The data presented in this study are available on request from the corresponding author. The data are not publicly available due to need approval from the affiliated institution’s DRB (Data review board) is required for disclosure or export.
